# The impact of the SARS-CoV-2 pandemic on the mental health of hemodialysis patients in Lebanon

**DOI:** 10.25122/jml-2020-0165

**Published:** 2021

**Authors:** Chadia Beaini, Mabel Aoun, Chebli El Hajj, Ghassan Sleilaty, Nicole Haber, Ghassan Maalouf, Eliane Abi Rached

**Affiliations:** 1. Department of Nephrology, Faculty of Medicine, Saint-Joseph University, Beirut, Lebanon; 2. Department of Nephrology, Bellevue Medical Center, Mansourieh, Lebanon; 3. Department of Nephrology, Saint-George Hospital, Ajaltoun, Lebanon; 4. Department of Biostatistics, Faculty of Medicine, Saint-Joseph University, Beirut, Lebanon; 5. Department of Cardiovascular Surgery, Hôtel-Dieu de France Hospital, Beirut, Lebanon; 6. Administrative Department, Bellevue Medical Center, Mansourieh, Lebanon; 7. Department of Psychology, Bellevue Medical Center, Mansourieh, Lebanon

**Keywords:** hemodialysis, COVID-19, mental health

## Abstract

Hemodialysis is a necessary treatment for end-stage kidney disease patients. It imposes undergoing three sessions of dialysis per week in a specialized center. Amid the SARS-CoV-2 pandemic, precautionary measures were mandatory in all dialysis facilities and may have negatively impacted patients’ well-being. This study aimed to uncover the scale of this effect. We performed a cross-sectional study of all patients undergoing chronic hemodialysis in two dialysis units (one urban and another rural). Patients with Alzheimer’s disease were excluded. Patients filled a questionnaire including information on socio-demographics, factors related to the dialysis facility, and the impact of the COVID-19 epidemic on their mental health. A total of 72 patients responded. Their median age was 70 (60.79) years. Of them, 68% were males, 71% were married, and 10% were living alone. Following the pandemic, 35% felt more anxious, with a higher incidence of anxiety in the rural unit (p=0.021). Half of them felt very limited in their relationships, and 29% were isolated from their families. In total, 98% of patients were satisfied with the staff support. The imposed preventive measures were perceived as very strict in 27% of the surveyed patients. The majority of the urban group were bothered for not eating during the session, and they felt significantly more stress than the rural group (p=0.001). The SARS-CoV-2 pandemic increased anxiety among hemodialysis patients from a rural setting. Stress was more prevalent in the urban group and most probably related to limitations in eating during sessions. The majority were satisfied with staff support.

## Introduction

The Severe Acute Respiratory Syndrome Coronavirus 2 (SARS-CoV-2) outbreak was declared as a pandemic by the World Health Organization in March 2020 and has spread widely around the world. Coronavirus Disease 2019 (COVID-19) is a severe illness of international concern [1]. Primary importance has been given to physical health and treatment of symptoms. However, it is essential to look at the psychological health ensued by the viral infection, isolation, restricted social activities, lockdown, and forged news; these factors can generate stress, anxiety, and episodes of depressive reactions [2]. A large survey done in Italy during the epidemic peak found high levels of post-traumatic stress disorder, depression, anxiety, insomnia, and perceived stress in the general population [3]. In an internet survey of adults (n >1400) from the United States (US) in April 2020, psychological distress, including depression, hopelessness, and nervousness, was present in 14 percent of surveyed individuals [4]. Another recent survey suggested that the prevalence of depressive symptoms in the US was more than 3-fold higher during COVID-19 compared to the pre-COVID-19 pandemic era [5].

Hemodialysis (HD) patients were found to be at high risk of developing COVID-19 and exhibited a high rate of mortality [6, 7]. In this perspective, there was a need to draw specific recommendations to take appropriate infection control measures to prevent COVID-19 spread in this vulnerable population and guarantee continuity of care [8]. Understandably, this new situation could lead to psychological distress due to fears of infection inside the hospital since they still had to come to the hospital to receive their treatment. Like chronic cancer patients, dialysis patients had to follow all the restrictive measures imposed by the hospitals to prevent them from contracting the virus [9].

In addition, patients undergoing HD are confronted with many chronic physical and psychosocial constraints. Fatigue, exhaustion, lack of appetite, sleeping problems, change in body image, and sexual dysfunction are seen together with physiological complications due to HD treatment [10]. Anxiety and depression, which are common psychiatric disorders among HD patients, can be triggered by these factors [11, 12]. Interestingly, health care professionals tend to focus on the biological dimension of the disease or the technical issues related to dialysis and underestimate symptoms from the mental sphere [13]. An underestimated or untreated anxiety and depression may lead to a diminished quality of life [14].

To the best of our knowledge, the epidemic’s impact on the mental health of HD patients has not yet been evaluated. This study aimed to analyze the psychological impact of the COVID-19 pandemic on this frail population of HD patients and unveil the factors associated with poor mental health.

## Material and Methods

### Study design

This descriptive study has a cross-sectional design. It was conducted in the HD units of 2 hospitals in Lebanon: Bellevue Medical Center (BMC), a center close to the capital Beirut and Saint-George Ajaltoun Hospital (HSGA), a center located in the mountains. The study took place in the first week of July 2020, during the peak of the COVID-19 spread.

### Population

The study population consisted of all patients with chronic HD in the units of BMC and HSGA. The inclusion criteria for the study were patients with chronic HD who were willing to participate in the study. Patients with Alzheimer’s disease or another dementia were excluded.

### Questionnaire

The questionnaire consisted of four parts (available in the supplemental data file). The first part assessed the socio-demographic details of the patients (age, gender, marital status, educational level, the people with whom the patient lives, social interactions or problems with others), and the second part assessed the medical details (dialysis vintage, psychiatric problems before COVID-19: anxiety disorder, mood disorders such as depression, major depression, bipolar disease or psychotic disorders such as schizophrenia). In the third and fourth parts, the patient had to answer many questions concerning mental health compared to before the COVID-19 period.

This study’s questionnaire was extracted from the “Kidney Disease Quality of Life Instrument” (KDQOL) [15], and “Hospital Anxiety and Depression Scale” (HADS) [16]. Items relevant to the study’s objectives were selected. The questions were divided into four parts and were formulated in an easy way that can be understood by the patients since the assessment was not supervised by a psychologist.

### Statistical analysis

Continuous variables were reported as mean and standard deviation (SD) if normally distributed and median and interquartile (IQR) if skewed. Categorical variables were described as numbers and percentages. The Mann-Whitney U test was used to compare continuous variables such as age. Chi-Square and Fischer exact tests were used to compare categorical variables. Statistical analysis was performed using SPSS software (IBM Corp. Released 2019, SPSS Statistics for Windows Version 26.0, Armonk, NY).

## Results

### Baseline characteristics

Among the 80 patients screened, two patients were excluded for having Alzheimer’s disease, and 2 patients refused to give consent for the study. A total of 76 were included, but only 72 patients finally filled the questionnaire: 42 patients at BMC (urban center) and 30 patients at HSGA (rural center). The median age of patients was 70 (60.79) years, with 68% males; 71% were married, and 10% were living alone. A third of patients had university educational levels. Eighty percent of patients did not have any psychological problem before COVID-19. Only two patients had a social conflict. No patient from both centers suffered from COVID-19. There was no difference in the baseline characteristics between the two hospitals (Table 1).

**Table 1.** Baseline characteristics of patients.

		**Total n=72**	**BMC n=42**	**HSGA n=30**	**P-value**
**Age**		70 (60.79)	68 (58.79)	73 (63.76)	0.248 *
**Sex, n (%)**	**Male**	49 (68.1)	31 (73.8)	18 (60)	0.215 **
	**Female**	23 (31.9)	11 (26.2)	12 (40)	
**Educational level, n (%)**	**Illiterate**	4 (5.6)	1 (2.4)	3 (10)	0.560 **
	**Elementary**	16 (22.2)	9 (21.4)	7 (23.3)	
	**Secondary**	22 (30.6)	12 (28.6)	10 (33.3)	
	**University**	25 (34.7)	17 (40.5)	8 (26.7)	
	**Technical**	5 (6.9)	3 (7.1)	2 (6.7)	
**Familial situation, n (%)**	**Single**	4 (5.6)	1 (2.4)	3 (10)	0.315 **
	**Married**	51 (70.8)	29 (69)	22 (73.3)	
	**Widow**	11 (15.3)	7 (16.7)	4 (13.3)	
	**Divorced**	6 (8.3)	5 (11.9)	1 (3.3)	
Living mode, n (%)	**Living Alone**	7 (9.7)	5 (11.9)	2 (6.7)	0.255 **
	**With Siblings**	2 (2.8)	2 (4.8)	0 (0)	
	**Parents**	1 (1.4)	0 (0)	1 (3.3)	
	**Children**	15 (20.8)	9 (21.4)	6 (20)	
	**Spouse**	19 (26.4)	8 (19)	11 (36.7)	
	**All Family**	27 (37.5)	17 (40.5)	10 (33.3)	
	**Other**	1 (1.4)	1 (2.4)	0 (0)	
**Social conflict, n (%)**	**No**	68 (97.1)	38 (95)	30 (100)	0.503 ***
	**Yes**	2 (2.9)	2 (5)	0 (0)	
**Dialysis vintage, n (%)**	**Days**	1 (1.4)	0 (0)	1 (3.3)	0.483 **
	**Months**	13 (18.1)	8 (19)	5 (16.7)	
	**Years**	58 (80.6)	34 (81)	24 (80)	
**Psychological problems before COVID-19, n (%)**	**No**	57 (80.3)	34 (82.9)	23 (76.7)	0.513 **
	**Yes**	14 (19.7)	7 (17.1)	7 (23.3)	

* – Mann-Whitney U Test; ** – Pearson Chi-Square Test; *** – Fischer Exact Test.

### Patients’ psychology during COVID-19 epidemic

The impact of the pandemic on patients’ psychology is summarized in Table 2. Following the pandemic of COVID-19, 35% of patients were more anxious compared to the pre-epidemic period. Half of them were more limited in their relationships with others, and 29% were isolated from their families. Severe organic anxiety was noted in 5 patients. When asked about all psychological changes, 22% of patients felt that nothing has changed. Because of the pandemic, 20% were more worried about their health, 24% felt angry, 4% guilty, 24% sad, and 12.5% depressed. More than 50% of patients had insomnia and fatigue, 12% anorexia, and 8% difficulty in concentration at work. When comparing the two centers, more patients from the rural center were anxious compared with the urban center, with a significant difference (p=0.021).

**Table 2.** Impact of SARS-CoV-2 pandemic on patients' psychology.

		**Total n=72**	**BMC n=42**	**HSGA n=30**	**P-value**
**Compared to before COVID-19, you are more anxious, n (%)**	**No**	47 (65.3)	32 (76.2)	15 (50)	0.021 **
	**Yes**	25 (34.7)	10 (23.8)	15 (50)	
**Compared to before COVID-19, you are more limited in relationships, n (%)**	**No**	31 (43.1)	16 (38.1)	15 (50)	0.315 **
	**Yes**	41 (56.9)	26 (61.9)	15 (50)	
**Compared to before COVID-19, you have severe non-organic anxiety, n (%)**	**No**	69 (95.8)	41 (97.6)	28 (93.3)	0.567 ***
	**Yes**	3 (4.2)	1 (2.4)	2 (6.7)	
**Compared to before COVID-19, you have severe organic anxiety, n (%)**	**No**	67 (93.1)	38 (90.5)	29 (96.7)	0.393 ***
	**Yes**	5 (6.9)	4 (9.5)	1 (3.3)	
**Compared to before COVID-19, nothing has changed, n (%)**	**No**	56 (77.8)	31 (73.8)	25 (83.3)	0.338 **
	**Yes**	16 (22.2)	11 (26.2)	5 (16.7)	
**Feeling of Anger, n (%)**	**No**	55 (76.4)	32 (76.2)	23 (76.7)	0.963 **
	**Yes**	17 (23.6)	10 (23.8)	7 (23.3)	
**Feeling of Guilt, n (%)**	**No**	69 (95.8)	41 (97.6)	28 (93.3)	0.370 **
	**Yes**	3 (4.2)	1 (2.4)	2 (6.7)	
**Feeling of Sadness, n (%)**	**No**	55 (76.4)	32 (76.2)	23 (76.7)	0.963 **
	**Yes**	17 (23.6)	10 (23.8)	7 (23.3)	
**Feeling of Shame, n (%)**	**No**	72 (100)	42 (100)	30 (100)	-
	**Yes**	0 (0)	0	0	
**Feeling of Depression, n (%)**	**No**	63 (87.5)	38 (90.5)	25 (83.3)	0.366 **
	**Yes**	9 (12.5)	4 (9.5)	5 (16.7)	
**Insomnia, n (%)**	**No**	35 (48.6)	23 (54.8)	12 (40)	0.217 **
	**Yes**	37 (51.4)	19 (45.2)	18 (60)	
**Anorexia, n (%)**	**No**	63 (87.5)	37 (88.1)	26 (86.7)	0.857 **
	**Yes**	9 (12.5)	5 (11.9)	4 (13.3)	
**Fatigue, n (%)**	**No**	36 (50)	24 (57.1)	12 (40)	0.151 **
	**Yes**	36 (50)	18 (42.9)	18 (60)	
**Lack of concentration ,n (%)**	**No**	66 (91.7)	37 (88.1)	29 (96.7)	0.390 ***
	**Yes**	6 (8.3)	5 (11.9)	1 (3.3)	
**Worrying about one's health, n (%)**	**No**	58 (80.6)	34 (81)	24 (80)	0.920 **
	**Yes**	14 (19.4)	8 (19)	6 (20)	
**Isolated from family during the epidemic, n (%)**	**No**	51 (70.8)	29 (69)	22 (73.3)	0.693 **
	**Yes**	21 (29.2)	13 (31)	8 (26.7)	

** – Pearson Chi-Square Test; *** – Fischer Exact Test.

### Factors related to the dialysis unit

Most of the patients did not have any difficulty reaching the unit during the lockdown. They considered that the dialysis team provided enough information and good communication concerning the COVID-19 and the precautionary measures. Seventy-five percent of the patients were very satisfied with the emotional support given by the dialysis team. All patients considered that the hospital took enough precautionary measures to control the infection and protect the patients from the spread of the virus. Almost one-third of the patients considered these measures strict and threatening their daily habits in the dialysis unit. There was no significant difference between the urban and the rural centers concerning all these parameters (Table 3).

**Table 3.** Factors related to the dialysis unit during the COVID-19 epidemic.

			**Total n=72**	**BMC n=42**	**HSGA n=30**	**P-value**
**Difficulty to reach the dialysis unit, n (%)**	**No**		67 (93.1)	39 (92.9)	28 (93.3)	0.990 ***
	**Yes**		5 (6.9)	3 (7.1)	2 (6.7)	
**Medical problems during the epidemic, n(%)**	**No**		52 (74.3)	31 (75.6)	21 (72.4)	0.763 **
	**Yes**		18 (25.7)	10 (24.4)	8 (27.6)	
**Emotional support at the dialysis unit, n(%)**	**Is Not At All Satisfactory**		0	0	0	0.592 **
	**Not Satisfactory**		1 (1.4)	1 (2.4)	0	
	**Neutral**		0	0	0	
	**Satisfactory**		17 (23.9)	11 (26.2)	6 (20.7)	
	**Very Satisfactory**		53 (74.6)	30 (71.4)	23 (79.3)	
**Unit team gives enough information and communicate well about COVID-19, n (%)**	**No**		2 (2.9)	2 (5)	0	0.506 ***
	**Yes**		67 (97.1)	38 (95)	29 (100)	
**The hospital established prevention and infection control measures in order to protect you from COVID-19, n (%)**	**No**		0	0	0	-
	**Yes**		71 (100)	42 (100)	29 (100)	
	**If yes, do you think these measures are very strict and threaten your habits?**	**No**	49 (73.1)	29 (70.7)	20 (76.9)	0.577 **
		**Yes**	18 (26.9)	12 (29.3)	6 (23.1)	

** – Pearson Chi-Square Test; *** – Fischer Exact Test.

### The degree of stress

After implementing the strict precautionary measures in the dialysis unit, almost half of the patients were bothered from wearing a facemask during the session, 35% were bothered by being prevented from eating during the session, and 8% were mostly bothered from not seeing their families. Half of the patients felt stressed. When comparing the two centers, patients from the urban center were significantly more bothered by not eating during the session and were significantly more stressed than those from the rural center (Table 4).

**Table 4.** What bothers patients the most and the degree of stress perceived

		**Total n=72**	**BMC n=42**	**HSGA n=30**	**P-value**
**What bothers you the most during COVID-19 period: Wearing a facemask, n (%)**	**Total**	61 (100)	35 (100)	26 (100)	0.432 **
	**No**	34 (55.7)	18 (51.4)	16 (61.5)	
	**Yes**	27 (44.3)	17 (48.6)	10 (38.5)	
**What bothers you the most during COVID-19 period:** **Being prevented from eating during the dialysis session, n (%)**	**Total**	61 (100)	35 (100)	26 (100)	<0.001 **
	**No**	40 (65.6)	15 (42.9)	25 (96.2)	
	**Yes**	21 (34.4)	20 (57.1)	1 (3.8)	
**What bothers you the most during COVID-19 period:** **Not seeing my family, n (%)**	**Total**	61 (100)	35 (100)	26 (100)	0.382 ***
	**No**	56 (91.8)	31 (88.6)	25 (96.2)	
	**Yes**	5 (8.2)	4 (11.4)	1 (3.8)	
	**Yes**	1 (1.6)	1 (2.9)	0	
**To what extent do you feel stressed, n (%)**	**Not stressed at all**	38 (55.9)	17 (40.5)	21 (80.8)	0.001 **
	**Somewhat stressed**	12 (17.6)	8 (19)	4 (15.4)	
	**Moderately stressed**	14 (20.6)	14 (33.3)	0	
	**Very much stressed**	3 (4.4)	2 (4.8)	1 (3.8)	
	**Extremely stressed**	1 (1.5)	1 (2.4)	0	

** – Pearson Chi-Square Test; *** – Fischer Exact Test.

## Discussion

This study, conducted in two different in-hospital HD centers in Lebanon, affirmed the negative impact of the COVID-19 pandemic on the mental health of HD patients. Two-thirds of patients felt psychological changes caused by the pandemic, 45% were stressed, and 35% were more anxious after the spread of the coronavirus. Our findings are consistent with previous studies that showed the unfavorable psychological effect of COVID-19 on mental health in general [4, 5, 17]. Most of these studies have focused on the mental health of the general population or healthcare staff. Few studies analyzed the effect on chronic patients, such as patients with cancer, but our study is the first to specifically examine the psychological influence of COVID-19 on HD patients [9, 18]. Moreover, our study showed that only a few patients were not affected by the epidemic. Before COVID-19, 20% of the surveyed patients suffered from psychological problems. During the epidemic, many negative feelings were perceived, and the emotions experienced by dialysis patients reflected psychosomatic and mood disorders; even anger was felt by 30% of them. Many patients also had symptoms of anxiety disorder like fatigue and insomnia, and 23% felt sadness. Anxiety disorder has been shown to be directly associated with poor quality of life in end-stage kidney disease patients [19].

Interestingly, there was a geographical contrast between our two surveyed groups (urban and rural) concerning the impact of COVID-19 on the dialysis patient’ s psychology. The urban population showed significantly more stress but less anxiety. This can be explained by the cultural and subjective environment of the individual. In the urban center, the patients were more bothered by wearing a mask, being prohibited from eating food during the session, and being isolated from their families, explaining the higher degree of stress. On the other hand, living in the city, around hospitals equipped to receive and treat COVID-19 patients may decrease the level of anxiety. It is noteworthy that our finding is not consistent with other previous studies regarding these aspects [20, 21]. In a survey of 187 HD patients, anxiety, sleep disorders and depression were uniformly found independent of the socio-demographic profiles of patients [20]. In another study, patients from urban areas exhibited more anxiety than those from rural areas [21].

Our study showed that 20% of patients were more worried about their health after the COVID-19 pandemic despite the staff support. Knowing that emotional and physical security are strongly linked in dialysis patients [22], a multidisciplinary team of nephrologists, mental health professionals, and nurses is essential in helping patients to manage the challenging demands after any acute stress (physical or emotional stress). A healthy environment for dialysis patients is mandatory, based on good communication, education, and emotional support. In our survey, 98% of patients were satisfied with the emotional support given by the staff, revealing the appropriate emotional and medical security provided by both centers’ environments. This high level of satisfaction with staff is aligned with other HD cohorts in Lebanon [23]. There is a clear relationship between the care satisfaction and quality of life of dialysis patients, making the healthy interaction between the patient and health care providers crucial in any dialysis unit [24]. Another essential factor that plays a significant role in HD patients’ psychology is social support [25, 26]. It has been demonstrated that low perceived social support leads to poor quality of life in HD patients [26]. On the other hand, social support from family seems to be an effective therapy for anxiety, depression, and other psychological disorders in HD patients [25]. Many dialysis patients rely on their family members for their daily activities and medical care. Since more than two-thirds of our patients were not isolated from their family members, we can consider that this factor may have alleviated the prevalence of more severe psychological disorders in these patients. Finally, it is noteworthy that home hemodialysis could be an alternative to improve the mental health of elderly patients and reduce their risk of infection [27]. Unfortunately, in our context, home hemodialysis is not reimbursed by third-payers and was not proposed to patients.

This study has several limitations. First, the sample size is small and is not representative of all hemodialysis patients; therefore, the results might not depict the overall scenario of the whole hemodialysis population. The study was proposed to many hemodialysis centers, but these centers were busy implementing precautionary measures to reduce the spread of the virus. Second, a self-filled questionnaire might not always be able to reflect authentic levels of psychological impact in patients; the psychologists regularly present in the dialysis units were restricted from the hospital during the lockdown period. Third, the study was done at the beginning of the extensive spread of the virus and did not reflect the real fear of patients with the massive spread after July in Lebanon.

## Conclusion

To our knowledge, this is the first study that surveyed the impact of the COVID-19 pandemic on the mental health of chronic HD patients. Our findings are valuable because they provide information on the prevalence of psychological disorders in the HD population. They also deciphered associated risk factors which, in turn, could help identify the most vulnerable groups of patients. Screening and treating mental disturbances in HD patients are essential steps to improve the quality of life in this susceptible population.

## Acknowledgments

### Ethical approval

The approval for this study was obtained from the Ethics Committees (International Review Board) of the two hospitals: Bellevue Medical (approval ID: BMC 3/2020) and Saint-George Ajaltoun Hospital (approval ID: RCEM/1/2020).

### Consent to participate

The participants entered the study voluntarily and data were collected anonymously.

### Conflict of interest

The authors declare that there is no conflict of interest.

## Supplementary Material


