# Supplementary material for: The impact of the SARS-CoV-2 pandemic on the mental health of hemodialysis patients in Lebanon
Source: J Med Life. 2021 Jul-Aug;14(4):523–30. doi: 10.25122/jml-2020-0165 (PMC8485366; doi:10.25122/jml-2020-0165)
Supplement: Supplementary file 1 [file JMedLife-14-523-s001.pdf]

Supplemental data file. Questionnaire for hemodialysis patients during the COVID-19 pandemic.

Sociodemographic data

Date of birth	• dd//mm/yyyy
Gender	• Male<br>• Female<br>• Other
Highest level of education	• None<br>• Primary school or less<br>• Secondary school<br>• University degrees (Bachelor/Master/Doctorate)<br>• Technical studies
Marital status	• Single<br>• Married<br>• Widowed<br>• Divorced
At home	• Living alone<br>• Living with siblings (brothers and sisters)<br>• Living with parents<br>• Living with children<br>• Living with spouse<br>• Living with all the family<br>• Other (in dorms, rental with friends etc)
Social interactions<br>*Are you suffering from problems with others?*	• Yes<br>• No<br>If yes:<br>○ Problems with relatives (parents, children, spouse etc)<br>○ Problems with colleagues at work<br>○ Other

Medical data

Duration on dialysis	• Days<br>• Months<br>• Years<br>• Lifetime
Did you have Psychiatric problems before Covid-19?	• Yes<br>• No<br>If yes:<br>○ Anxiety disorders<br>○ Mood disorders (depression: major depression/bipolar)<br>○ Psychotic disorders (schizophrenia)

Psychological data

Compared to before covid-19 period (you can choose more than one answer)	• You are more worried about your kidney disease<br>• You are more limited in certain activities or relationships<br>• You are feeling an acute stress that does not interfere with your physical integrity<br>• You are feeling an acute stress that interferes with your physical integrity<br>• None
Emotional problems<br>*Do you feel?* (You can choose more than one answer)	• Anger<br>• Guilt<br>• Sadness<br>• Shame<br>• None<br>• Other
Do you have any of the following symptoms? (You can choose more than one answer)	• Feeling nervous<br>• Feeling down<br>• Trouble sleeping<br>• Decreased appetite<br>• Decreased energy<br>• Difficulty concentrating<br>• Thinking too much about your health which is preventing you from working

Concerning COVID-19 safety measures

Have you isolated yourself from your family for safety reasons?	• Yes<br>• No<br>If yes, what are you feeling about this situation? (You can choose more than one option)<br>○ Nothing at all, feeling fine<br>○ Feeling lonely<br>○ This situation is negatively interfering with my family relationships
Have you had difficulty reaching the dialysis center because of the lockdown?	• Yes<br>• No<br>If yes, you felt (You can choose more than one option):<br>○ Nervous<br>○ Stressed<br>○ Angry<br>○ Aggressive<br>○ Hopeless
Have you faced any medical problems during this period?	• Yes<br>• No<br>If yes, please specify
How do you describe the emotional support of the dialysis staff? (Please choose one answer)	• Very unsatisfying<br>• Unsatisfying<br>• Neutral<br>• Satisfying<br>• Very satisfying
Does the dialysis staff provide you healthy communication and good education about the COVID-19?	• Yes<br>• No
Does the hospital take safety measures to protect you from COVID-19 infection?	• Yes<br>• No
What bothers you the most during this period?	• Wearing the facemask<br>• Being prevented from eating during dialysis sessions<br>• Not seeing your family<br>• Other
To what extent do you feel stressed?	• Not stressed at all<br>• Somewhat stressed<br>• Moderately stressed<br>• Very much stressed<br>• Extremely stressed
